# From Scaffolds to Complex Systems: Functional Biomaterials in Regenerative Medicine

**DOI:** 10.3390/jfb17010051

**Published:** 2026-01-19

**Authors:** Cristian Scheau, Andreea Cristiana Didilescu, Constantin Caruntu

**Affiliations:** 1 Department of Physiology, The “Carol Davila” University of Medicine and Pharmacy, 050474 Bucharest, Romania; 2 Department of Radiology and Medical Imaging, “Foisor” Clinical Hospital of Orthopaedics, Traumatology and Osteoarticular TB, 021382 Bucharest, Romania; 3 Department of Embryology, Faculty of Dentistry, The “Carol Davila” University of Medicine and Pharmacy, 050474 Bucharest, Romania; 4 Department of Dermatology, “Prof. N.C. Paulescu” National Institute of Diabetes, Nutrition and Metabolic Diseases, 011233 Bucharest, Romania

Regenerative medicine stands at a crossroad between biology and materials science, where functional biomaterials are expected to interact with living tissues, guide repair, and restore functionality [1]. The advancements in material composition, biofabrication, and degradability all work towards the same objective: the design and implementation of regenerative systems capable of actively enhancing healing via interactions with the biological environment [2,3]. This Special Issue brings together diverse papers but with a unified concept, collectively reflecting an honorable ambition of creating biomaterials that do not simply fill defects but actively orchestrate regeneration.

Recent studies have challenged the long-standing paradigm of biomaterials as inert fillers. The systematic review by Ivanova et al. has provided a comprehensive overview of the effectiveness of traditional autografts, allografts, xenografts, and also synthetic materials in various biologic axes [4]. The authors have underlined the trade-off between biological activity and mechanical reliability, which was a recurrent theme in the papers of this Special Issue. This topic was also addressed by another team of authors, which explored biodegradable magnesium-based alloys where Mg-Nd and Mg-Zn are not only studied for their mechanical properties, but also for the kinetics of degradation and tissue responses in vivo [5]. The materials dissolution is reframed here from a limitation to a therapeutic parameter that must be very carefully adapted to synchronize the resorption of the scaffold with bone regeneration. This supports the idea that future biomaterials must be designed as temporally adaptive constructs that evolve together with the surrounding supported tissue [6]. This concept was further expanded by Yu et al. in their paper on the properties of epigallocatechin gallate to retain antioxidant activity when properly processed, thereby bridging molecular bioactivity and scalable biomaterial fabrication [7].

Beyond the topic of bulk composition, multiple authors have emphasized the important role of micro- and nano-architecture in defining tissue interactions. The electrospinning of collagen and collagen-glycosaminoglycan mats demonstrated how minute variations in the morphology of fibers and biochemical composition can influence stem cell adhesion, viability, and proliferation [8]. This reinforces the concept that cells respond not only to physical but also biochemical signals, a concept that resonates with recent work on functional biomaterials [9]. The relevance of architectural design is equally critical on the macrostructural scale, in the field of 3D bioprinting. The extensive review on soft tissue bioprinting by Timofticiuc et al. showed that the current challenge goes beyond material selection and relies on the spatial organization of multiple cell types, bioinks, and mechanical gradients within the construct [10]. The authors show that anatomical fidelity should be doubled by functional integration and that advancements in the field of bioprinting should address not only resolution, but also strategies for vascularization and innervation. These concepts align with recent papers, which have also signaled the necessity of consistent progress in this direction [11,12].

Vascularization appears as a current limiting factor in various classes of materials in different tissues. The study by Tojo et al. demonstrated that embedding vascular networks within the engineered constructs leads to improved angiogenesis and accelerates the wound-healing process [13]. The authors show that host-graft interactions are guided by the microvascular architecture and this underlines the broader theme that the capacity to recruit vasculature is indispensable for clinical translation, be it on a micro- or nano- scale, or even on larger, more complex structures such as entire limbs [14]. Vascular healing may be targeted and controlled on a cellular level by specific biomaterials; this was shown in the paper by Zhu et al. which inhibited vein graft restenosis via developing a chlorogenic acid–strontium bioresorbable stent [15].

Alongside vascularity, preservation and modulation of neural integrity are recognized as highly important factors for functional regeneration and integration [16,17]. Radecka et al. have synthesized the emerging approaches for biomaterial-based neural protection and shown how the control of the microenvironment can promote regeneration and mitigate secondary injury [18].

Overall, across all tissue applications, we are witnessing a maturation of the field of biomaterials, which leads to adaptive, transient, and biologically instructive platforms. Viewed collectively, the manuscripts in this Special Issue reflect and actively advance the coherent evolution and common theme documented in the recent literature: there is a continued pursuit of engineering more sophisticated biomaterials and integrating them into regenerative strategies, appropriate to the complexity of human biology. Advancements are rapid across all domains, and we are eagerly anticipating solid breakthroughs in near-future studies.

## Data Availability

No new data were created or analyzed in this study. Data sharing is not applicable to this article.
